# Community Participation in Primary Healthcare in the South Sudan Boma Health Initiative: A Document Analysis

**DOI:** 10.34172/ijhpm.2022.6639

**Published:** 2022-04-12

**Authors:** Loubna Belaid, Iván Sarmiento, Alexander Dimiti, Neil Andersson

**Affiliations:** ^1^CIET-PRAM (Participatory Research at McGill), Department of Family Medicine, McGill University, Montreal, QC, Canada.; ^2^Grupo de Estudios en Sistemas Tradicionales de Salud, Universidad del Rosario, Bogotá, Colombia.; ^3^Department of Reproductive Health, Ministry of Health, Juba, South Sudan.; ^4^Centro de Investigación de Enfermedades Tropicales, Universidad Autónoma de Guerrero, Acapulco, Mexico.

**Keywords:** Community Participation, South Sudan, Healthcare Access, Document Analysis, Primary Healthcare

## Abstract

**Background:** Community participation is central to primary healthcare, yet there is little evidence of how this works in conflict settings. In 2016, South Sudan’s Ministry of Health launched the Boma Health Initiative (BHI) to improve primary care services through community participation.

**Methods:** We conducted a document analysis to examine how well the BHI policy addressed community participation in its policy formulation. We reviewed other policy documents and published literature to provide background context and supplementary data. We used a deductive thematic analysis that followed Rifkin and colleagues’ community participation framework to assess the BHI policy.

**Results:** The BHI planners included inputs from communities without details on how the needs assessment was conducted at the community level, what needs were considered, and from which community. The intended role of communities was to implement the policy under local leadership. There was no information on how the Initiative might strengthen or expand local women’s leadership. Official documents did not contemplate local power relations or address gender imbalance. The policy approached households as consumers of health services.

**Conclusion:** Although the BHI advocated community participation to generate awareness, increase acceptability, access to services and ownership, the policy document did not include community participation during policy cycle.

## Background

**Key Messages**

** Implications for policy makers**

- Policy-makers need to create a safe space for enabling participation. Policy-makers need to pay attention to local power dynamics and hierarchies.
- Policy-makers need to consider gender imbalances to increase women’s participation.
- Policy-makers and planners need to discuss with communities to see how they see service delivery, what obstructs the access to healthcare.
- Building capacities at all levels are necessary for meaningful participation.
- Community representatives need to be engaged in planning, implementing, evaluating policies to increase ownership.

** Implications for the public**

Community participation in health policies contributes to the local relevance of health and social interventions. Community participation can positively affect the health and well-being of a population. As the Boma health policy is currently formulated, community participation does not fully engage communities in the policy cycle. The role of households is reduced to use the services, and the Boma health committees are expected to implement the policy. This study identifies research gaps and implications for further action to increase meaningful community participation in primary healthcare in South Sudan. The Boma health planners need to create a safe space to discuss service delivery with communities and what obstructs healthcare access. They need to ensure financial support and building capacities to increase participation.

The Alma Ata Declaration recognized community participation as central to primary healthcare.^1^ More recently, community participation was featured on the roadmap to achieve the Sustainable Development Goals and as a core tenet in right-based approaches to health and social determinants for health.^2,3^ Engaging communities in health policies positively affect social and health outcomes and ensures contextual relevance of interventions to local needs.^4,5^ Yet community participation pathways to health improvement are not well understood, given the complex influences of contextual factors.^6^

 A body of literature explores the concept of community participation and its meanings.^5-9^ There is no consensus on the definition of community participation in healthcare. The umbrella term incorporates a range of concepts and activities, such as consultation, involvement, mobilization, or empowerment. In this study, we understood community participation as “a process by which people are enabled to become actively and genuinely involved in defining the issue of concerns to them, in making decisions about the factors that affect their lives in formulating and implementing policies, in planning, developing, and delivering services and in taking action to achieve change.”^10^

 Identifying *whom *to engage, *why, when, *and *how *community participation should happen is essential for meaningful community participation. These elements are highly contextual,^11^ and policy documents often fail to assess community participation in health planning and implementation.^5,12,13^ The lack of evidence on participation in conflict settings is even starker.^14^ Ongoing conflicts, violence, forced displacement of population, weakened healthcare systems, disruption of social cohesion and community ties, and heavy presence of international organizations might influence the design, implementation, and evaluation of health programs in conflict settings and the level of community involvement along the process.^14^

 In 2016, the Government of South Sudan launched the Boma Health Initiative (BHI) to foster primary healthcare and support community participation. The BHI policy aims to reduce preventable diseases and deaths by increasing the equitable utilization of services and community participation in health activities, thereby ensuring the sustainability of the community health structure and services.^15^

 “The BHI is intended to replace the current fragmented community health services (preventive, curative, and promotional) that non-governmental organizations provide with funding from different donors.”^16^

 The Initiative has three main policy goals: (*i*) develop community health structures as a standard component of the national health system, (*ii*) increase access to quality health promotion, disease prevention, and selected curative services through community engagement (*iii*) provide leadership for the implementation of the BHI through inter-sectoral collaboration and community participation.^16^

 Through the BHI, trained community health workers are responsible for delivering a standard integrated package of promotional, preventive, and selected curative health services at the Boma level, the basic unit of local government. These services focus on child health, communicable disease control, safe motherhood, health management information systems and surveillance. In addition, community health workers receive training on disease surveillance and reporting service delivery data and vital statistics.^15^

 This study assessed the extent to which the South Sudan Ministry of Health addressed community participation in its health policy formulation and provided recommendations to policy-makers.

###  Study Setting 

 The comprehensive peace agreement signed between the Government of Sudan and the Sudan People’s Liberation Movement/Army in 2005 ended one of the longest conflicts in the African continent (1956–1972 and 1983–2005).^17,18^ South Sudan gained independence in 2011, making this country the world’s youngest nation. It had an estimated population of 11 million in 2019, with about 80% living in rural and remote areas.^19^ More than 80% of the South Sudan population lived below the poverty line in 2016^16^ and was agro- pastoralist. The literacy rate is low (34.52%), and it is even lower for women (28.86%) when they are compared to men (40.26%).^20^ The maternal mortality ratio is 789 maternal deaths per 100 000 live births, and the infant mortality rate is 60 deaths per 1000 live births. The utilization of healthcare services is low, the rate for facility-based delivery is 21%, and only 2.6% of children had all nine recommended vaccinations.^21^

 State, county, payam, and boma are the country’s administrative units. Boma is the smallest unit, composed of small villages and hamlets. Primary teaching hospitals, state hospitals, county hospitals, primary healthcare centers and healthcare units constitute the healthcare system’s levels. Primary healthcare units are the lowest level facilities and provide preventive, promotional, and curative services.

 The Ministry of Health identified and prioritized a list of essential health services, known as the Basic Package of Health and Nutrition Services (BPHNS). This package should be affordable and accessible to most of the population at the primary and secondary healthcare levels (Box 1). The South Sudan government and multilateral donor agencies (Health Pooled Fund, HPF) and the World Bank fund the BPHNS. The South Sudan HPF is a multi-donor funding mechanism that currently includes six donors: the United Kingdom, Canada, the European Union, Sweden, the United States, and GAVI. This funding mechanism operates in 23 geographic areas in eight of the ten former states by contracting non-governmental organizations.^22^ The HPF expanded community health services based on BHI structures.^23^

**Box 1. Maternal, Newborn, Reproductive, and Child Health Services in the Basic Package of Health and Nutrition Services in South Sudan**

**Maternal and newborn health**

- Safe motherhood/ essential obstetric care
- Antenatal care
- Delivery care
- Care for newborn
- Post-partum care
- Information, education, communication

**Reproductive health and family planning**

- Post abortion care
- Modern contraceptive methods
- Screening and treatment for sexually transmitted diseases
- Screening and treatment for HIV
- Information, education, communication

**Child health/integrated essential child health**

- Community based child survival
- Expanded programme on immunization
- Essential nutrition action
- Home treatment of malaria, diarrhoea, and pneumonia

## Methods

###  Context of the Study and Study Design 

 This study was part of a larger research project examining health policies and systems landscapes to improve maternal and child health in South Sudan and assess how the South Sudan Ministry of health incorporated equity, gender, and community participation in its health policies and programs. The overall project methods included a scoping review, key informant interviews, and a health policy analysis. The scoping review and key informant interviews results were published elsewhere.^21^ This paper describes only the health policy analysis based on a document analysis.^24^

 We used appropriate theory (Rifkin framework) and method (documentary analysis) to examine the BHI policy.^25^ We described the positionality of the authors of this manuscript.^24^ The Authors of this study include a male South Sudanese policy-maker from the National Ministry of Health and two male researchers based in Canada, a geographer and public health specialist and an epidemiologist with three decades of experience in participatory research. The lead author is a female Muslim Arab anthropologist specializing in gender and equity who worked in a participatory community-based program to improve maternal and child health in Torit County, South Sudan and met with national policy-makers who designed the BHI. The authors account for decades of experience in participatory research in fragile settings in Colombia, Mexico, Nicaragua, Guatemala, Nigeria, Eritrea, northern Uganda, and Pakistan.

###  Data Collection Methods and Data Analysis 

 Our document analysis^25^ focused on the BHI policy. We consulted other documents related to the Boma Health policy and the South Sudan health system to provide background context and supplementary data. The policy documents came from the South Sudan Ministry of Health and its international partners and academic peer-reviewed publications (Supplementary files 1, 2 and 3). A deductive thematic approach analyzed the BHI policy document.^26^ The deductive thematic categories are the indicators of the community participation conceptual framework developed by Susan Rifkin and colleagues.^27^ This framework is based on over 100 case studies on health programs in low-middle income countries.^27^ Other studies using this framework found it straightforward to assess health programs’ participation in these settings and remote communities in high-income countries.^12,28,29^

 The framework includes five indicators for participation: (1) *needs assessment: *to what extent communities were involved in identifying and defined their health needs and designing the intervention, (2) *community organization:* to what extent the program integrates or collaborates with pre-existing community structures, (3) *program management:* community’s capacity to make decisions about the programs’ direction and development, (4) *leadership development:* the inclusiveness and representativeness of all community interests groups, and (5) *resources mobilization: *communities’ ability to mobilize and contribute resources towards programs.

 Rifkin defined equity as “community ability to deal with the problems of the very poor” and recognized it as the sixth dimension of the framework. Although not incorporated as an indicator for assessing the continuum of participation, she considered equity a significant factor when analyzing participation.^27^ We, therefore, included equity in our application of this framework.

## Results

###  Needs Assessment 

 The BHI designers consulted international partners. The Ministry of Health staff visited Cuba, Ethiopia, Rwanda, and Turkey to understand the community health systems in those countries to inform the development of the BHI.

 The document mentioned that planners included inputs from communities without giving many details on how the need’s assessment was conducted at the community level and what needs were considered, and from which community:

 “*Through consultations with countries in the African region and beyond, we have finally conceptualized the BHI. It has been tailored to our local circumstances and shaped by local experience with invaluable inputs from partners in the field as laid down in this implementation guideline*”(The BHI, 2016).

 The BHI designers focused on delivering packages dependant on external resources without considering if this was creating additional needs or contrasting those packages with the needs expressed by the communities.

 The content of the package is based on the situational health analysis of the country and suggested delivering a package of services:

 “*The top ten or so diseases in South Sudan are preventable communicable diseases (…). The high infant and maternal mortality rates in the Country are largely due to preventable conditions. It is imperative that the health system targets and engages individuals, households, and communities to communicate health risks and related costs for health action to improve health outcomes” *(The BHI 2016).

 “*To achieve this policy objective, the government shall: define and provide the guiding principles for the delivery of the basic package of health and nutrition services [BPHNS] for health promotion, disease prevention, treatment, and rehabilitation to improve health, reduce mortality and morbidity among all communities in South Sudan. (…) The BHI shall provide a service package drawn from the BPHNS that is aligned with the country’s disease profile” *(The BHI, 2016).

###  Community Organization and Program Management 

 The county-payam-boma administrative system takes its roots in Sudan’s colonial history. Since colonial time, this system has remained unclear, resulting in several interpretations on how it should be implemented.^22^

 In implementing the policy, the BHI designers proposed delivering the services through this hierarchal administrative system. The BHI designers rely on a top-down community health structure at the boma level. This top-down community structure is composed of three components: (*i*) the Boma administration, composed of a chief and administrator, (*ii*) the Boma health committee, composed of the Boma administration, the health facility in-charge, headteachers, representatives of respectable personalities, women and youth group members, individuals with disabilities, and interest groups and (*iii*) the Boma health teams composed of three community health workers. The policy document did not describe how to ensure that the most vulnerable groups, including women, will be members of the Boma health committees. These community structures’ roles were to implement the policy and deliver their services at the Boma level. Still, they had not a clear role in the decisions about the program directions and development:

 “*Boma administration is responsible for implementing all government and development programs at the Boma level; generates development needs that feed into the bottom-up planning process. It follows that the Boma administration is responsible for the functionality of the community health system, the BHI, as a government structure to support the health sector development program” *(The BHI, 2016).

 “*The BHI carries out health promotion, provide basic curative care, and support community management information system (community-based surveillance and vital statistics)” *(The BHI, 2016).

 In the policy document, BHI designers conceptualize households only as consumers of health services. The health system expectation is households improving health-seeking behaviours and adopting, maintaining, or restoring health. The document did not describe any household’s participation related to the policy’s design, implementation, and evaluation.

 “*Communities are active consumers of health services. The ultimate responsibility to address the health determinants *&* health risks and find alternatives to mitigate the effects of health inequities lies in the hands of individual households with government setting enabling environment. Health education aims to create awareness to stimulate appropriate action to promote, maintain, or restore health” *(BHI, 2016).

###  Leadership Development 

 The initiative designers explained the composition of the Boma committee responsible for implementing the policy. The policy relied on local leadership to ensure the policy’s implementation. In the policy document, “communities nominate the Boma Health Teams through a competitive process into the public sector, appraise the BHT for rewards, and recommend replacing an absentee or non-performing BHT member to the recruiting authority and reviewing program reports and resource use for accountability.” Yet, neither the policy document details which “communities” refers to and how this governance should occur.

###  Resource Mobilisation

 The government and its development partners funded the policy with documented intent to put community health workers on the national public payroll, support training, and fund the tools. The government expected mobilisation of additional funds from different levels of the health system:

 “*The Government of the Republic of South Sudan shall finance salaries of the community health workers (staff), the recurrent budget of the Payam health office, BHT and home health promotors acquire tools and equipment, and procurement of medical and health commodities” *(The BHI, 2016).

 The policy document expected that the local government generate their fund to support the policy; however, the local government might not have the capacity to mobilize resources because it is not built upon local strengths but as an external definition of priorities and means.

 “*The Local governments: These funds may come from locally generated funds, conditional grants for capacity building in local governments, or discretionary funds available to the counties from any source” *(The BHI, 2016).

###  Concentrating on the Needs of the Poor

 The policy did not distinguish between different vulnerable groups of the population. The overall goal was to ensure that every household could benefit from the healthcare services package, that services were affordable to all groups:

 “*To ensure universal health coverage for all communities through effective, affordable, and comprehensive delivery of the BPHNS. Universal coverage with basic health services ensures equitable access to health services by the population. It encompasses geographical coverage, coverage of the population groups, and a service package that is well-aligned with the health needs of the population who should access services without financial hardship at the point of care” *(The BHI, 2016).

## Discussion

 From the policy document, it is difficult to assess to what extent community problems were defined and how the BHI designers evaluated the community’s needs. The solutions deliver packages dependent on external resources without considering if this was creating additional needs or contrasting those packages with the needs expressed by the communities. Community structures were nonetheless intended to implement the resulting policy, relying on local leadership that, up to the release of the policy, had no experience to support this role. The policy document offered no information on how to strengthen or expand the influence of local leadership, much less consider the role of women. The populations were viewed as consumers for health services designed outside their Boma.

###  Participation From Need’s Assessment to Policy Implementation

 Despite its prominence in the policy document, participation remained limited in how the roles of community structures and households were framed. Households were viewed only as consumers of health services, with community leaders the supposed – but unfunded and untrained – implementers of policies. This finding aligns with several recent systematic reviews on community participation.^4,14,30-32^ A 2015 research synthesis reported that 95% of initiatives they reviewed engaged communities in implementing interventions, and only 18% involved them in identifying and defining problems.^4^ A 2020 systematic review on community participation in humanitarian settings reported limited evidence on involvement of communities in framing issues or designing solutions.^14^ A 2021 scoping review describing patient public engagement strategies for health system improvement in sub-Saharan Africa found that participation was limited to consultation but not involved in the final decision-making process. None of the studies reported shared leadership and concluded that the patient public strategies are characterized by tokenism rather than participation.^30^ Another systematic review found that communities did not identify health problem research at the problem definition stage but rather learned about a health problem pre-determined by researchers, which is the same case for the BHI.^32^ Designers focused the issue on access to primary healthcare services.

###  Political Organization and Leadership

 The current political situation in South Sudan does not favour community participation, being a volatile state with a fragile peace process.^33^ Continued political conflicts rooted in ethnic tensions reduce the sustainability of peace efforts.^34^ Besides the current national conflict, inter-communal clashes are common.^34,35^

 This political context obstructs community participation in South Sudan in at least three ways. First, it decreases the trust relationship between communities and the government, leading to underuse health services.^36^ Second, crystallization of power relationships into political factions does not facilitate equal engagement of all community groups.^34,35^ Although BHI planners relied on pre-existing community structures to implement the policy, politicization of these structures can exclude some community segments from the program. The segments could be the ethnic groups that do not belong to the dominant groups or have a privileged relationship with the ethnic group in a power position. South Sudan is a home country for more than 20 ethnic groups and the Dinka is the largest group, followed by the Nuer. A body of evidence reported an association between politics and ethnicity.^34,35,37,38^ Third, the ongoing conflict led to insecurity obstructing communities’ free participation in health programs.^14^

 Power dynamics can affect the policy implementation and outcomes by preventing some groups from accessing the services. A 2020 evaluation of community health worker programs in South Sudan, for example, reported that favouritism in the recruitment of community health workers impeded equity in health programs.^39^

 Systematic reviews showed how studies failed to examine the gender imbalances in leadership in community-based health programs.^4,14^ In many settings, leaders are mostly men, and literate community health workers are men. The 2020 study in South Sudan reported challenges in recruiting literate community health workers, especially among women. The same study described how community members prefer female community health workers. They also mentioned that presence of women in Boma health committees was limited despite the requirement that one-third of the members were women.^31^

###  Resource Mobilisation

 Proponents of the Alma Ata approach to primary healthcare have long warned against policy shifts that presume spontaneous community participation in healthcare without funding provisions.^40-42^ The BHI is predominately dependent on external funding and expects the lower levels of government to contribute financially to the implementation of the policy. Evidence from Mali, Ethiopia, and Guinea showed how financial constraints, including short-term donor funding, are an essential barrier to community participation and sustainability.^43-45^ In contrast, studies from the Democratic Republic of Congo and Sierra Leone found that individual financial or in-kind compensation triggered community participation.^46-48^

 In South Sudan, resource mobilization is not only a challenge specific to implementing the BHI policy, but it is also a national challenge to function the entire country, despite its abundant natural resources. South Sudan has large oil reserves. They are located on the border with Sudan. Oil is garnering the source of nearly the entire basis of the accounting for of South Sudan’s government revenues and its gross domestic product.^49,50^ In 2012, the Government of South Sudan suspended oil production and export, representing 98% of the country’s revenue. This suspension meant a revenue loss of about $650 million each month, with negative impacts on the health and other sectors.^51^ Since then, the budget allocated to health has decreased year after year.^51^ In 2019, the total domestic allocation was 1.9%. As a result, the Ministry of Health cannot carry out its activities, improve facilities infrastructures, or pay its human resources without international donors and foreign governments.^52,53^ Up to 2021, community health workers were not on the national payroll. They were not trained because of a lack of funding. South Sudan remains a humanitarian actor-led country in which United Nations agencies and other humanitarian actors define priorities, choose interventions to be implemented and deliver health services, reducing participation at all health system levels.^36,53^

###  Primary Healthcare Services Utilization and Health Equity 

 The BHI’s rationale was that community participation would arise to change community attitudes and actions underlying poor health and the low use of health services. They assumed that making services available at the periphery of the health system would increase uptake and thus coverage of health services. Summarised in the scoping review results after 2017, there were no reports of change in the utilization of health services coverage through the Initiative.^21^ A 2020 South Sudan study, based on a desk review and secondary analysis of intervention coverage reported levels of antenatal care, institutional delivery, and childhood vaccination remained low through 2017.^54^ The lack of impact of the BHI policy on coverage of essential health services could be explained by the very novelty of the policy, its partial implementation in the ten states involved in the Initiative and significant existing gaps in the health system. Human resources shortages at all health system levels, lack of medicines and supplies, perceived poor quality of care, and chronic under-funding are the main bottlenecks identified in the health system.^21^

 The BHI is not an oriented pro-equity policy but toward universal health coverage through expecting households to pay for affordable health services. This raises considerable challenges to how health improvement can be made, knowing that 80% of the population lives below the poverty line and most of the infrastructure (water, sanitation, roads, hospitals, schools) are almost inexistent.

###  Research Gaps and Policy Implications for Future Actions

 While a body of evidence is emerging on community participation in fragile settings, gaps in knowledge remain on the optimum role and scope of community engagement, best methods to engage communities and sustain it, and measuring the impact of their engagement on health outcomes. More research is also needed on exploring power dynamics within community structures and how to reduce their effects.

 BHI planners need to create a safe space for enabling participation to discuss how communities service delivery and what obstructs access to healthcare. They need to pay attention to local power dynamics and hierarchies and consider gender imbalances to increase women’s participation. They need to ensure financial support and building capacities to increase participation.

###  Limitations 

 To the best of our knowledge, this is the first health policy analysis of the BHI based on in-depth documentary analysis, review of publications of previous empirical work in South Sudan; however, this study has significant limitations.

 We did not conduct fieldwork with communities to assess their perceptions of their participation levels in the initiative. We did not rank the five indicators of the Rifkin framework with the 1-5 scale during the key informant interviews to draw the spider grams. The spider-grams and the empirical qualitative study would have undoubtedly enriched and increased the study’s internal validity. This is a unique case study, so the transferability of results to other conflict-affected countries may be limited.

## Conclusion

 Its policy documents show the BHI intended community participation as a strategy to generate awareness, to increase the acceptability of and access to services. The policy document did not engage Boma level stakeholders in its formulation or provide for community participation throughout the policy cycle. The current political and economic situation and bottlenecks within the health system hinder community participation development.

## Ethical issues

 We obtained ethical approval and consent for publication from the ethical committee of the Ministry of Health, Republic of South Sudan (Moh/ERB 472018).

## Competing interests

 Authors declare that they have no competing interests.

## Authors’ contributions

 LB designed the study and conducted the document review. LB, IS, and NA participated in the intellectual content analysis, interpretation of the findings. LB and NA drafted the manuscript. LB, NA, and IS reviewed manuscript for important intellectual content. LB, AD, IS, and NA read and approved the final manuscript.

## Supplementary files


Supplementary file 1. List of Policy Documents Consulted.

Supplementary file 2. List of Published Literature Consulted.

Supplementary file 3. Maternal, Newborn, Reproductive, and Child Health Services in the Basic Package of Health and Nutrition Services in South Sudan.
