# Supplementary material for: Community Participation in Primary Healthcare in the South Sudan Boma Health Initiative: A Document Analysis
Source: Int J Health Policy Manag. 2022 Apr 12;11(12):2869–75. doi: 10.34172/ijhpm.2022.6639 (PMC10105198; doi:10.34172/ijhpm.2022.6639)
Supplement: Supplementary file 1 — List of Policy Documents Consulted. [file ijhpm-11-2869-s001.pdf]

Article title: Community Participation in Primary Healthcare in the South Sudan Boma Health Initiative: A Document Analysis

Journal name: International Journal of Health Policy and Management (IJHPM)

Authors' information: Loubna Belaid[1]*, Iván Sarmiento[1,2], Alexander Dimiti[3], Neil Andersson[1,4]

[1]CIET-PRAM (Participatory Research at McGill), Department of Family Medicine, McGill University, Montreal, QC, Canada.

[2]Grupo de Estudios en Sistemas Tradicionales de Salud, Universidad del Rosario, Bogotá, Colombia.

[3]Department of Reproductive Health, Ministry of Health, Juba, South Sudan.

[4]Centro de Investigación de Enfermedades Tropicales, Universidad Autónoma de Guerrero, Acapulco, Mexico.

(*Corresponding author: Email: lbelaid@ciet.org)

Supplementary file 1. List of Policy Documents Consulted

Number	Policy document title	Year of Publication	Type of documents
1	Basic Package of Health and Nutrition Services in Primary Health Care	2011	Implementation guidelines
2	The Family Planning Policy	2013	Health policy
3	The National Health Policy (2016-2025)	2016	Health strategic plan
4	The National Health Strategic Plan (2016-2025)	2016	Health strategic plan
5	South Sudan adolescence sexual and reproductive health strategic plan	2018	Health strategic plan
6	The Community Health System in South Sudan: The Boma Health Initiative	2016	Implementation guideline
7	Reproductive health strategy (2018-2022)	2018	Health strategic plan
8	South Sudan National Emergency Obstetric and newborn care assessment	2014	Nationwide health assessment
9	Rapid facility survey	2013	Nationwide health assessment
10	Rapid assessment of the status of Reproductive Maternal Newborn Adolescent Child Health and nutrition services in South Sudan	2014	Report
11	Health Situation analysis for the National Health Policy Update	2014	Report
12	Every newborn action plan	2018	Health strategic plan
13	Midwifery training policy	2018	Implementation guideline
14	Maternal Infant and young child nutrition strategy (2017-2025)	2017	Health strategic Plan and Implementation guideline
