# Supplementary material for: Community Participation in Primary Healthcare in the South Sudan Boma Health Initiative: A Document Analysis
Source: Int J Health Policy Manag. 2022 Apr 12;11(12):2869–75. doi: 10.34172/ijhpm.2022.6639 (PMC10105198; doi:10.34172/ijhpm.2022.6639)
Supplement: Supplementary file 2 — List of Published Literature Consulted. [file ijhpm-11-2869-s002.pdf]

Article title: Community Participation in Primary Healthcare in the South Sudan Boma Health Initiative: A Document Analysis

Journal name: International Journal of Health Policy and Management (IJHPM)

Authors' information: Loubna Belaid[1]*, Iván Sarmiento[1,2], Alexander Dimiti[3], Neil Andersson[1,4]

[1]CIET-PRAM (Participatory Research at McGill), Department of Family Medicine, McGill University, Montreal, QC, Canada.

[2]Grupo de Estudios en Sistemas Tradicionales de Salud, Universidad del Rosario, Bogotá, Colombia.

[3]Department of Reproductive Health, Ministry of Health, Juba, South Sudan.

[4]Centro de Investigación de Enfermedades Tropicales, Universidad Autónoma de Guerrero, Acapulco, Mexico.

(*Corresponding author: Email: lbelaid@ciet.org)

Supplementary file 2. List of Published Literature Consulted

References	Study design and data collection	Settings	Population and sample size	Reproductive Maternal Newborn Adolescent Child health
Berendes and al. Assessing the quality of care in a new nation: South Sudan's first national health facility assessment. Tropical Med Int Health. 2014;19(10):1237–48.	health facility assessment<br>Cross-sectional survey<br>Infrastructure<br>Material and supplies<br>Health workers<br>Patient records	Nationwide	14-16 health facilities in each of 10 States (n=156 randomly selected facilities)	Children consultation<br>Antenatal care
Elmusharaf and al. (2017) Social and traditional practices and their implications for family planning: a participatory ethnographic study in Renk. South Sudan Reprod Health. 2017;14(1):1	Participatory Ethnographic evaluation and research<br>In-depth interview<br>Workshop	Renk County, Northern Upper Nile State	14 women selected from villages of Renk County	Family planning (family size)
Kane (2016) Social norms and family planning decisions in South Sudan. BMC Public Health.;16(1):1183	Qualitative exploratory study<br>FGD (n=5)<br>Individual interviews (n=44)	Wau County, Wau County, State of Western Bahr el Ghazal (Fertit community: rural and urban)	Community members (male and female)<br>Traditional healers<br>Traditional birth attendants<br>Health facility personnel<br>State SRH managers<br>NGO representation	Family planning (social norms shaping birth spacings, childbearing Marriage)

Kane and al. (2018) Too afraid to go fears of dignity violations as reasons for non-use maternal health services in South Sudan. Reprod Health.;15(1):51	Qualitative exploratory study FGD (n=5) Interviews (n=44)	Same as Kane (2016)		Maternal health (perception of the use of services for childbirth)
Lawry and al (2017) A mixed-methods assessment of maternal, newborn and child health barriers in Gogrial west, South Sudan. Reprod Health. 2017;14(1):12.	Cross-sectional study Randomized household survey quantitative, qualitative interviews (n=72	Gogrial West,Warrap State	Pregnant women who had children less than five years of age (n=860) Men (=144) Qualitative interviews (n=72)	Contraception<br>Facility based-deliveries<br>Antenatal care<br>Malaria (mosquito nets)<br>DPT3 immunization<br>Gender-based violence
Mugo and al. (2016) Factors associated with different types of birth attendants for home deliveries: an analysis of the cross-sectional 2010 South Sudan household survey. Glob Health Action. 2016;9:29693	Cross-sectional 2010 South Sudan household survey	National	2767 mothers who gave birth within two years before the survey and delivered their babies at home	Maternal health (childbirth)
Mugo and al. (2015) Prevalence and risk factors for non-use antenatal care visits: analysis of the 2010 South Sudan household survey. BMC Pregnancy Childbirth. 2015;15(1):68.	Analysis of the 2010 South Sudan household survey (a nationally representative, stratified, cluster sample survey)	National	3504 women aged 15-49 years who had given birth within two years preceding the survey	Antenatal care
Mugo and al. (2015) Maternal and Child Health in South Sudan. Priorities for the Post-2015 Agenda;5(2) Sage Open	Literature review on MNCH in South Sudan	National	Na	Maternal and child health
Mugo and al. (2018) "The system here isn't on patients' side"- perspectives of women and men on the barriers to accessing and utilizing maternal healthcare services in South Sudan. BMC Health Serv Res	A qualitative study (in-depth interviews)	Juba County, Equatoria State)	Women with children aged less than three months, 10 in each type of health care facility who had given birth either at home or in a health facility(n=30), husbands (n=15)	

Mugo and al. (2018) Determinants of neonatal, infant and under-five mortality in a war-affected country: analysis of the 2010 household health survey in South Sudan. BMJ Glob Health	Secondary analysis of the 2010 South Sudan household survey	National	8125 singleton, live birth, under-five children born in South Sudan within five years before the 2010 South Sudan Household Survey	
Palmer and al. (2016) Building the nation's body: the contested role of abortion and family planning in post-war South Sudan. Soc Sci Med. 2016;168:84–92.	Ethnography study of public health interventions (document analysis, observation, key informant interviews (n=54)	Juba	Health managers from the MOH, Ministry of Gender,<br>Local and international NGO staff and UN agencies nurse and local women's organization Journalists	
Sami and al. (2018) Understanding health systems to improve community and facility level newborn care among displaced populations in South Sudan: a mixed-methods case study. BMC Pregnancy Childbirth. 2018;18(1):325.	Cross-sectional descriptive study of facility-based deliveries (clinical observation, exit interviews, health facility assessment, direct observation of midwife time use)	Displacement camps: Maban Gendrassa Kaya, Juba, and Malakal	Mother-newborn pairs who sought services and birth attendants who provided delivery services between April and June 2016 in 5 health facilities	
Scott and al. (2013) An assessment of attitudes toward gender inequitable sexual and reproductive health norms in South Sudan: a community-based participatory research approach. Confl Heal. 2013;7(1):1–8	Community-based participatory research using quota sampling	Aweil (Northern Bahr El Ghazal), Wau (Westen Bahr El Ghazal), Lainya, Morobo, Ronyi (Central Equatoria), Malakal (Upper Nile State)	N= 680 respondents, n=352 female, n= 326 male	Gender norms regarding sexuality and reproductive health
Izudi and al. (2017) Early postnatal care use by postpartum mothers in Mundri East County, South Sudan. BMC Health Serv Res. 2017;17(1):442	Cross-sectional study (survey with random sampling)	Mundri East County (Western Bahr El Ghazal)	13 (one regional referral hospital, two county hospitals, 10 PHCCs) postpartum mothers who had a live birth were 15 -49 years old, 8-14 days post-delivery that, attended PNC clinics	Postnatal care

			(to receive immunization, contraception, growth monitoring services)	
Kane and al. (2016) Social norms and family planning decisions in South Sudan. BMC Public Health. 2016;16(1):1183.	Qualitative descriptive study (FGD, n=5; Interviews, n=44)	Kane and al, 2016, 2018)	Gender norms related to reproductive health	
Sami and al. (2017) You have to take action": changing knowledge and attitudes towards newborn care practices during the crisis in South Sudan. Reproductive health matters. 2017;25(51):124–39	A cross-sectional study based on self-reported questionnaire and interviews	IDP	Health providers Traditional birth attendants Community health workers	
Wilunda and al. (2016) Barriers to institutional childbirth in Rumbek North County, South Sudan: a qualitative study. PLoS One. 2016;11(12)	Qualitative study FGD (n=14) Interviews (n=12_	Rumbek County	Women (n=169) and men (n=45) Community leaders Health providers County health department staff	Antenatal care
Jones and al. (2015) Feasibility of health systems strengthening in South Sudan: a qualitative study of international practitioner perspectives. BMJ Open. 2015;5(12):1–9.	Qualitative study	Nationwide	International health (technical advisor, consultant, senior managers, supervisors) from international, local organizations, health providers (n=20)	Primary healthcare (MNCH)
Myake and al (2017) Sami and al. (2018) Understanding health systems to improve community and facility level newborn care among displaced populations in South Sudan: a mixed-methods case study. BMC Pregnancy Childbirth. 2018;18(1):325	Scoping review Case study using mixed methods (FGD, n=19), in-depth interviews (n=7), observation of health facilities readiness, documents	South Sudan IPD, hospital (n=1), PHCC (n=4) CH programs (n=4)	Newborn care	Human resources Midwifery

Kane and al. (2018) Too afraid to go: fears of dignity violations as reasons for non-use maternal health services in South Sudan. Reprod Health. 2018;15(1):51	Exploratory qualitative study (FGD, n=4), in-depth interviews (n=44)	Wau County	Married women, 18-35 years old, unmarried women, 18-35 years old, men (less and above 35 years old) Health providers	Childbirth
Kane and al. (2019) You have a child who will call you "mama": understanding adolescent pregnancy in South Sudan. Glob Health Action. 2019;12(1)	An exploratory qualitative study (individual interviews)	Wau county	Female and male youth (in-school, out of school, with children and no children)	Reproductive health for youth
