# Supplementary material for: Community Participation in Primary Healthcare in the South Sudan Boma Health Initiative: A Document Analysis
Source: Int J Health Policy Manag. 2022 Apr 12;11(12):2869–75. doi: 10.34172/ijhpm.2022.6639 (PMC10105198; doi:10.34172/ijhpm.2022.6639)
Supplement: Supplementary file 3 — Maternal, Newborn, Reproductive, and Child Health Services in the Basic Package of Health and Nutrition Services in South Sudan. [file ijhpm-11-2869-s003.pdf]

**Article title:** Community Participation in Primary Healthcare in the South Sudan Boma Health Initiative: A Document Analysis

**Journal name:** International Journal of Health Policy and Management (IJHPM)

**Authors' information**: Loubna Belaid[1]*, Iván Sarmiento[1,2], Alexander Dimiti[3], Neil Andersson[1,4]

[1]CIET-PRAM (Participatory Research at McGill), Department of Family Medicine, McGill University, Montreal, QC, Canada.

[2]Grupo de Estudios en Sistemas Tradicionales de Salud, Universidad del Rosario, Bogotá, Colombia.

[3]Department of Reproductive Health, Ministry of Health, Juba, South Sudan.

[4]Centro de Investigación de Enfermedades Tropicales, Universidad Autónoma de Guerrero, Acapulco, Mexico.

(*Corresponding author: Email: lbelaid@ciet.org)

**Supplementary file 3.** Maternal, Newborn, Reproductive, and Child Health Services in the Basic Package of Health and Nutrition Services in South Sudan

**Maternal and newborn health**
- Safe motherhood/ essential obstetric care (EOC)
- Antenatal care
- Delivery care
- Care for newborn
- Post-partum care
- Information, education, communication

**Reproductive health and Family Planning**
- Post-abortion care
- Modern contraceptive methods
- Screening and treatment for sexually transmitted diseases
- Screening and treatment for HIV
- Information, education, communication

**Child Health/ Integrated essential child health (IECHC)**
- Community-based child survival
- Expanded programme on immunization (EPI)
- Essential nutrition action (ENA)
- Home treatment of malaria, diarrhoea, and pneumonia
